# Quality Assessment and Physicochemical Characteristics of Commercial Frozen Vegetable Blends Available on the Polish Market

**DOI:** 10.3390/foods15020224

**Published:** 2026-01-08

**Authors:** Joanna Markowska, Anna Drabent, Natalia Grzybowska

**Affiliations:** Institute of Agriculture and Food Biotechnology, State Research Institute, 02-532 Warsaw, Poland; anna.drabent@ibprs.pl (A.D.); natalia.grzybowska@ibprs.pl (N.G.)

**Keywords:** frozen vegetables, vegetable blends, quality assessment, freezing technology

## Abstract

Frozen vegetables are increasingly valued for their nutritional stability and year-round availability. This study provides a comprehensive assessment of twenty commercially available frozen vegetable blends obtained from retail markets in Poland. Analyses included physicochemical parameters, instrumental measurements of texture, color (CIEL*a*b*), and evaluation of technological quality. The pH values ranged from 4.40 to 7.46, total acidity from 0.034 to 0.322 g/100 g, and dry matter content from 5.02 to 42.97%. The observed variability was mainly attributable to vegetable type and remained consistent with values reported for fresh produce, indicating that industrial freezing largely preserves chemical characteristics. Texture differed markedly between vegetable types, with hardness values ranging from 6 to nearly 100 N, while color parameters remained within typical ranges for blanched and frozen vegetables, suggesting effective pigment stability and enzyme inactivation. In contrast, substantial variability was observed in technological quality. Mechanical fragmentation exceeded acceptable limits in 30% of samples, and complete clumping of vegetable pieces (100%) was observed. Additional defects included frostbite and color deviations, and health-condition defects were observed. These results highlight considerable heterogeneity in frozen vegetable blends and emphasize the need for stricter control of raw materials, processing conditions, and cold-chain management to ensure consistent quality and consumer safety.

## 1. Introduction

Vegetable crops constitute a vital part of the human diet owing to their dense nutritional profile and diverse bioactive constituents. Their increased production and consumption have the potential to improve both food security and overall nutritional quality. According to the EUROSTAT, harvested production of vegetables in the European Union reached the volume of 62,342.10 thousand tones of the total area of 1929.53 ha as of the year 2024 [1]. Contemporary food trends, focused on increasing the share of convenient products with high nutritional value while reducing food waste, have contributed to a growing interest in frozen vegetables as an all year long available alternative to fresh produce. Freezing technology, considered one of the most effective methods of food preservation, prevents rapid deterioration of fresh produce, significantly slowing down the adverse biochemical and microbiological processes that affect the quality of fresh vegetables. Europe holds 34.51% of the global frozen vegetable market revenue in 2024, driven by strong consumption habits, strict safety standards, and advanced cold-chain logistics. Growing demand for convenient, healthy, and sustainable foods, along with the rise of plant-based and clean-label preferences, continues to support increasing consumption of frozen vegetables [2].

Vegetables are perishable and mostly seasonal goods. Their delicate structure and high water activity make them susceptible to microbial spoilage and oxidative deterioration. A wide range of methods has been developed to prolong their postharvest usefulness: pasteurization, sterilization, and drying (all before-mentioned applied with or without the use of preservatives), as well as low temperature storage (chilling and freezing). The desiccation process lowers the water activity level to a point where microbial growth and enzymatic activity are significantly reduced. The side effects are irreversible structural changes and a considerable loss of nutrients. Thermal methods of food preservation effectively eradicate microorganisms; however, they also change sensory properties such as color, flavor, and texture, in addition to a vitamin loss [3,4].

Freezing technology, considered one of the most effective methods of food preservation, prevents rapid deterioration of fresh produce, significantly slowing down the adverse biochemical and microbiological processes that affect the quality of fresh vegetables. Freezing is defined as subjecting food to temperatures below 0 °C, although internationally the reference value is set at −18 °C. It has been established that at the reference temperature, most of the water is in the form of ice crystals. That phenomenon impedes the proliferation of microorganisms and biochemical reactions, thereby allowing for prolonged storage, provided that the cold chain is properly maintained [4,5,6].

However, the final quality of the product is highly dependent on several factors that have an effect on potential freeze damage. Among them are product-related determinants like an inherent resistance of a raw material and its stage of maturity, a postharvest initial quality and health, a time lapse between harvesting and processing (pretreatment), and then the cold chain comprising cooling, freezing method and time, storage, and thawing conditions. In addition, pretreatment stages, i.e., blanching, influence quality by affecting nutrient levels, albeit the loss is minimal if the temperature and time are optimized for the particular vegetable type and size [4,5,7].

This article presents an overview of the quality of frozen vegetable blends based on cross-sectional analyses of products available on the Polish market.

## 2. Materials and Methods

### 2.1. Materials

A total of twenty commercially available frozen vegetable blends were purchased from retail markets in Poland. The products were obtained from different producers and represented a broad range of product categories available to consumers, including (i) pan-fried vegetable blend, (ii) soup blend, and (iii) simple vegetable blends (two- or three-component blends such as peas and carrots or vegetable bouquets). This selection ensured that the sample set represented both basic and more complex consumer-oriented frozen plant products. The blends consisted of various types of vegetables, including root vegetables, fruit vegetables, and brassicas.

Table 1 presents 12 representative examples of the collected blends, while in total, 20 different blends from various producers were analyzed. For each variant, five independent batches were collected. Each sample was collected in its original commercial packaging. Detailed information was collected regarding the data presented on the wrapping. Among them, the producer declared composition, package weight, packaging material (e.g., low-density polyethylene (LDPE) film or unspecified foil), and expiry date (“best before”) (Table 1). The presented food information particulars were according to the mandatory requirements of EU Regulation No 1169/2011 on the provision of food information to consumers [8]. Simultaneously, it was observed that some producers did not provide complete information on the type of packaging material, which may reflect inconsistencies in labeling practices. All samples were stored under refrigeration (below −18 °C) until further analysis, in accordance with recommended food-quality preservation guidelines.

Prior to analysis, samples were thawed under controlled laboratory conditions (approx. 20 °C). For multi-component blends, the individual vegetable components were separated manually for each type of blend and batch. The results for vegetables of the same type (e.g., carrots, onions) but originating from different blends were averaged for all batches of a given blend. Then, based on the percentage content of a given vegetable in the blend, the quantities of the tested quality characteristics were calculated. For further physicochemical and instrumental analyses, the collected results, differentiated by vegetable, were averaged among the producer, blend type, and batch to obtain species characteristics for analysis. A total of 100 individual samples (20 blend variants × 5 batches) were analyzed, ensuring sufficient representativeness of both blend and batch variability. This approach allowed for a comparison of producers and product categories while taking into account the variability between blends and batches.

### 2.2. Standard Quality Evaluation

Each frozen product was evaluated according to the Polish Standard PN-A-78608 [9] “Deep frozen vegetable blend”, which defines the required visual, physical, and compositional characteristics of commercial frozen vegetable products. The evaluation was performed on thawed samples.

### 2.3. Physicochemical Analysis

All physicochemical parameters were determined following Polish Standards methods for fruit and vegetable products:

- Dry matter content: gravimetric method according to PN-A-75101-03 [10] (“Fruit and vegetable products—Preparation of samples and physical and chemical test methods—Determination of dry matter content by gravimetric method.”), using a moisture analyzer (MA40, Sartorius, Göttingen, Germany);
- Total acidity: by potentiometric titration according to PN-A-75101-04 [11] (“Fruit and vegetable products—Preparation of samples and physical and chemical test methods—Determination of total acidity.”), using pH meter (CP-505, ELMETRON Sp.j., Zabrze, Poland);
- pH: determined potentiometrically according to PN-A-75101-07 [12] (“Fruit and vegetable products—Preparation of samples and testing methods—Determination of pH by potentiometric method.”), using pH meter (CP-505, ELMETRON Sp.j., Zabrze, Poland).

All measurements were performed in triplicate, and results were collected in tables and presented in the following chapters as mean ± standard deviation.

### 2.4. Texture Measurement

The texture (hardness) of the vegetables was examined after bringing them to a temperature of approx. 5 °C (thawed) using a CT3 Texture Analyzer (Brookfield Ametek, Berwyn, PA, USA) equipped with Ottawa Cell for bulk compression. The maximum compression force (N) was recorded at a constant deformation speed of 1 mm/s (compression test). The vegetables were placed in the Ottawa Cell up to half its height (40–42 g) and then compressed with a 40 × 40 mm steel plate at a 1.0 mm/s over a distance of 10 mm. Each measurement was carried out in eight repetitions for each vegetable from the blend. The texture analysis of thawed samples was applied as an indirect indicator of freeze–thaw-induced water loss and structural degradation.

### 2.5. Color Measurement

The color parameters were determined in the CIE L*a*b* color space using a Konica Minolta CM-5 spectrophotometer (Tokyo, Japan). For the purpose of comparative analysis, the mean color parameters (L*, a*, b*, C*, h*) for each vegetable type were calculated based on all samples obtained from different blends and producers. This approach allowed for determining the typical color characteristics of frozen vegetables available on the market, regardless of their blend composition and whether the vegetables were at consumer maturity. Furthermore, color measurements were conducted using an aperture size of the spectrophotometer adapted to the dimensions of the vegetable to maximize the measured surface area and include any changes in color shades of the vegetable in the result. At least 12 repeated measurements were performed for each vegetable for a single blend, and the average values collected from all tested blends were used for further analysis.

### 2.6. Statistical Analysis

All data were processed using R software (v. 4.5.2). Descriptive statistics (mean ± SD, minimum–maximum) were calculated for all measured parameters.

## 3. Results and Discussion

### 3.1. Physicochemical Properties of Frozen Vegetables

The physicochemical parameters of vegetables affect not only their sensory attributes but also their stability and safety [13]. These parameters were determined after thawing and homogenization to ensure sample uniformity. The analyzed samples showed pH, total acidity, water content, and dry matter values typical for fresh vegetables (Table 2), confirming that chemical integrity was largely preserved during commercial freezing [14].

The differences in values between vegetables reflected the natural variability in organic acid composition among vegetable species. According to the literature, the fruit type of vegetables tends to have higher acidity (starchy fruit excluded) than leafy, tuber, and storage root type crops [14]. The total acidity was in the range of 0.034–0.322 g/100 g, with tomato showing the highest and corn the lowest values. The pH ranged from 4.40 to 7.46, with the lowest value observed in tomato and the highest in corn (Table 2). These findings remain consistent with reference values for fresh vegetables, where tomato typically exhibits a pH of 4.30–4.90, and corn reaches up to pH 7.50 [15]. This suggests that freezing and thawing do not substantially modify the acidity of vegetable tissues, a phenomenon previously reported for multiple frozen plant matrices [16,17].

Dry matter content varied primarily according to species-specific anatomy. Legume vegetables (e.g., beans, chickpeas) had the highest dry matter (up to 43%), while fruit vegetables (e.g., tomato, zucchini) showed the lowest values and the highest water content (over 90%). These patterns correspond to the structural composition of fresh vegetables, as legumes have dense, compact tissues rich in storage compounds that increase dry matter, whereas fruit vegetables contain porous parenchyma with large water-filled vacuoles, resulting in very high moisture content [18,19].

The texture (hardness) showed the greatest variability among all analyzed parameters. The broad range of values (6.00–99.00 N) resulted from the diversity of vegetable species present in the blends, each characterized by distinct mechanical properties. Another important source of variation was the significant structural diversity of different types of vegetables (i.e., grains, roots). Very soft fruit and vegetables differed noticeably from legumes, which are dense and compact in structure [20]. Together, the anatomical differences between plant types accounted for most of the observed variation. The freezing process additionally intensified variability within individual vegetable types. Ice-crystal formation can rupture cell walls and membranes, which reduces structural integrity and lowers turgor pressure. During freezing, ice crystallization occurs primarily in the extracellular space, increasing solute concentration and promoting water migration from the intracellular to the extracellular compartments [21]. As a result, vegetables from the same group may soften to different extents depending on their tissue compactness, cell size, and the proportion of structural components. Legumes, characterized by dense cell structures, showed the highest resistance to compression, while vegetables with delicate parenchyma (e.g., zucchini, eggplant) were the softest. Such variability is further influenced by moisture redistribution within the tissue: the release of intracellular water and the loss of structural integrity reduce turgor pressure, leading to softer textures, whereas lower free-water availability helps maintain firmness. The combined effects of cellular dehydration and mechanical stress caused by growing ice crystals lead to irreversible damage to cell walls and membranes, which becomes evident after thawing as reduced hardness. This trend corresponds with the well-established sensitivity of plant tissues to freeze-induced structural weakening [20,22]. Therefore, variability in hardness after thawing can be interpreted as a functional manifestation of water release from damaged cellular structures.

Overall, the analyzed frozen vegetables largely retained their chemical attributes at levels comparable to fresh produce, while structural integrity remained the most affected parameter. This confirms that freezing primarily influences texture, especially in high-moisture tissues, rather than chemical composition.

### 3.2. Color of Frozen Vegetables

The color is one of the most important sensory attributes determining consumer acceptance of fresh vegetables [23]. Based on this, customers form opinions about its quality, ripeness, and purchasing decisions. However, frozen vegetable packaging does not allow customers to make such assessments, so color remains an important quality indicator and should be evaluated in frozen vegetables. In the present study, the mean color parameters (L*, a*, b*) were determined for individual vegetables isolated from commercial frozen blends (Table 3). Due to the availability of only one producer of colored carrots (yellow, white, red, and purple), color measurements were not reported separately for these types. The presented color results refer to standard orange carrots, which are the ones most commonly used in frozen vegetable blends on the Polish market.

The brightest samples, characterized by the highest L* values, were potato, cauliflower, kohlrabi, and celery, reflecting their naturally light and weakly pigmented tissues. In contrast, paprika, eggplant, and broccoli exhibited lower L*, which is consistent with the presence of dark pigments such as flavonoids or anthocyanins in the case of purple varieties [24]. The a* coordinate, representing the red-green axis, varied from −10.20 (green pea) to +23.41 (tomato). The most intense red hues were observed for tomato, carrot, and paprika, which is consistent with their natural carotenoid (lycopene and beta-carotene) content [25]. Conversely, broccoli, green beans, and peas showed strong negative a* values, confirming the predominance of green tones associated with chlorophyll pigments. The b* values ranged between 14.54 and 40.64, and the highest yellowness was noted for carrot and corn, consistent with carotenoid-rich tissues [26,27]. Freezing and thawing can influence vegetable color through pigment degradation and structural changes, but in this study, the C* (chroma) and h (hue angle) values remained within ranges typical of fresh vegetables and within the normal color classification standards of the United States Department of Agriculture, USDA [28,29]. Color stability is supported by blanching, which inactivates oxidative enzymes such as polyphenol oxidase (PPO) and peroxidase (POD), and by low-temperature storage, which limits pigment degradation [30]. No severe discoloration was observed, suggesting that industrial pre-freezing processing was appropriate. Slight decrease in lightness and hue for broccoli, brussels sprouts, and paprika may result from partial chlorophyll and carotenoid degradation during blanching or frozen storage [30,31]. Overall, the color parameters demonstrate good visual quality and indicate that freezing, when combined with proper blanching and cold-chain maintenance, effectively preserves the characteristic color profiles of different vegetable types.

### 3.3. Quality

The quality assessment according to the Polish Standard PN-A-78608 “Deep frozen vegetable blend” revealed substantial variability among the 20 tested vegetable blends. The most decisive for consumer-perceived quality and overall compliance with the standard were selected for detailed assessment and used to visualize the quality of the blends (Table 4). Unlike physicochemical properties, which remained stable and did not pose safety risks, technological defects were frequent and, in some cases, severe. These defects are significant because they can indicate failures in manufacturing, raw material sorting, freezing kinetics, or cold-chain management, all of which are directly related to product quality or safety in particular cases.

**Figure 1:**
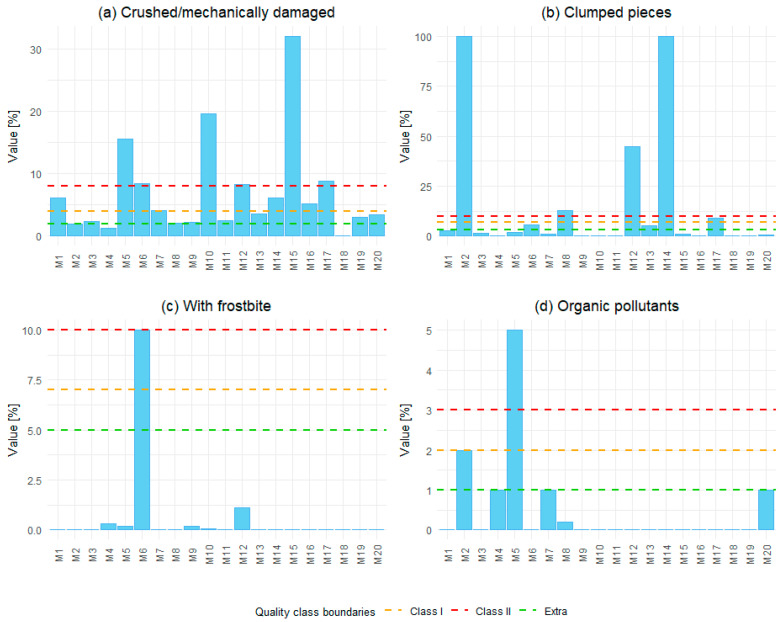
Quality assessment results for 20 frozen vegetable blends (M1–M20) based on four defect categories: (**a**) crushed/mechanically damaged, (**b**) clumped pieces, (**c**) with frostbite, and (**d**) organic pollutants. Dashed lines indicate the limit for Extra, Class I, and Class II quality classes according to PN-A-78608.

**Figure 2:**
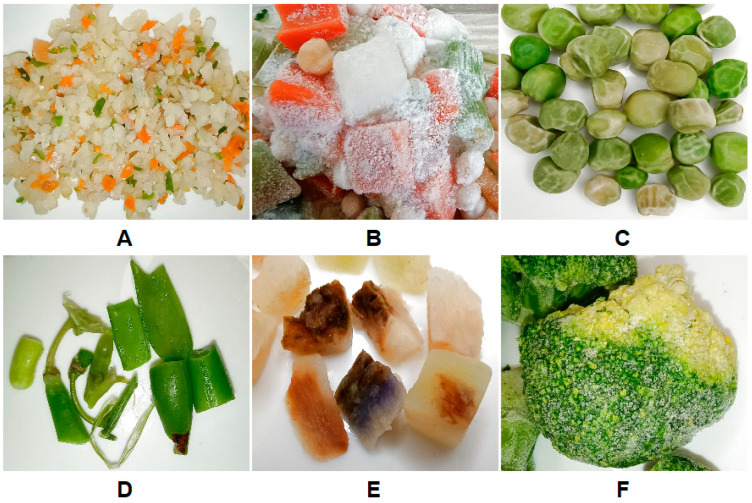
Examples of defects found in vegetable blends from different producers: (**A**) crushed/mechanically damaged vegetables, (**B**) clumped pieces, (**C**) green peas with frostbite, (**D**) residues of peel/inedible ends, (**E**) bad health condition, and (**F**) unripe broccoli.

The most common type of defect observed was mechanical damage and crushed vegetables, with 30% of the blends tested exceeding the acceptable limits for crushed vegetable fragments (Figure 1a). Especially in the case of ones containing vegetables with an irregular and tender structure, such as cauliflower (100% crushed in one of the blends) and onions, which were in the form of outgrades, i.e., small irregular fragments. One blend was also significantly crushed (over 30%), as shown in Figure 2A, where damaged pieces clearly illustrate the extent of mechanical breakdown. Mechanical loss may not pose a direct toxicological risk, but it reduces sensory quality, increases drip loss, and promotes infection by channeling and defense suppression [32]. The literature identifies mechanical fragmentation as a marker of poor handling during harvesting, trimming, or transport [33]. Therefore, high fragmentation is a quality defect and a potential indicator of deficient industrial processing controls.

Clumping of vegetable pieces was another critical defect affecting quality. In some blends, it occurred to a high degree, even in two cases accounting for 100% of the sample volume (Figure 1b), which indicates an incorrect freezing or storage process. Figure 2B visually confirms these extreme cases, showing tightly adhered pieces that compromise product homogeneity and consumer appeal. The main causes include the temperature fluctuations, which are one of the critical safety-relevant issues in frozen foods because they may enable microbial growth during partial thawing episodes, even if the product is refrozen. Such fluctuations degrade quality and may compromise safety if thawing was significant [13]. The clumping is therefore not only a technological fault but also an indicator of potential cold-chain disruptions.

In the case of frostbite, it appeared most frequently on cauliflower and green peas. For one sample, the value was 9.99%, which corresponds to the acceptable limit (10%) for the last quality Class III (Figure 1c). Visual evidence of frostbite on green peas can be seen in Figure 2C, highlighting the characteristic surface damage. The peas are pale with a white or greyish hue, lacking in juiciness after thawing. This defect is caused by surface dehydration through sublimation or temperature instability, resulting in white, dry, tough areas [34]. Although frostbite does not pose a microbiological risk, it can be used as an indicator of improper storage or prolonged exposure to excessively low or fluctuating temperatures. Thus, the frostbite is both a quality defect and a technological warning signal.

In addition to technological defects, various organic contaminants were also found, such as small fragments of plant origin, including grains, pits, seeds, and individual fragments of other plants. Their quantity was generally within acceptable limits. However, one blend exceeded the contamination limit for the lowest class (Figure 1d).

**Figure 3:**
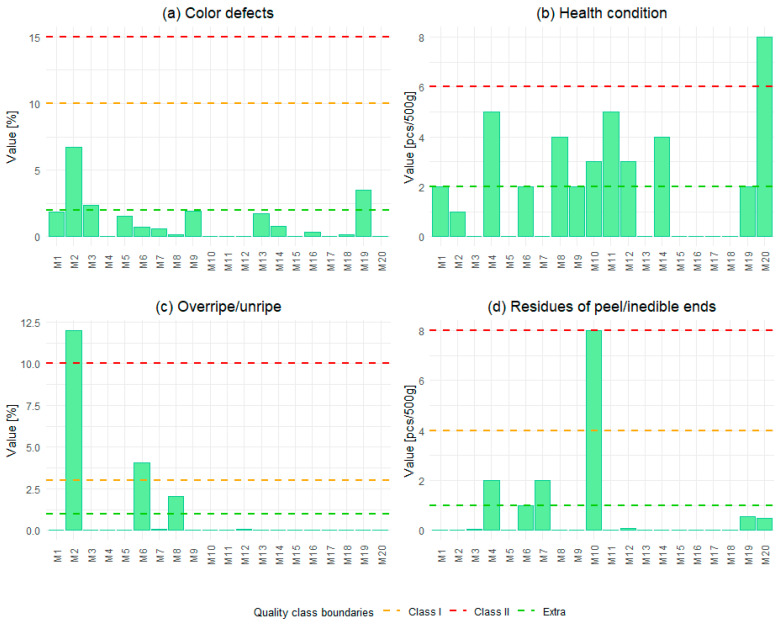
Quality assessment results for 20 frozen vegetable blends (M1–M20) based on four defect categories: (**a**) color defects, (**b**) health condition, (**c**) overripe/unripe, and (**d**) residues of peel/inedible ends. Dashed lines indicate the limit for Extra, Class I, and Class II quality classes according to PN-A-78608.

In regard to color and external appearance, typical defects were found, such as oxidative browning of celery, discoloration around the stalk and core of tomatoes, overly light color of leeks and green beans, and localized brown tips on pods. Many samples also contained fragments of cauliflower and broccoli stalks, which reduced the visual quality of the product. Most of the blends tested were classified as Class I in terms of color defects. Only three of them belonged to Class II (Figure 3a). An analysis of vegetable health showed that green peas, cauliflower, and carrots were most commonly damaged by disease. In several cases, the presence of brown or dark spots was observed, which may indicate early stages of spoilage. Individual deviations were also found in broccoli, chickpeas, eggplants, leeks, green beans, kohlrabi, potatoes, and onions. An example of such defects can be seen in the celery sample in Figure 2E. The samples fitted into different classes, but one exceeded the quality threshold (≤6.00 pcs/500 g) with a value of 8.00 (Figure 3b). The presence of these defects may be caused by poor harvesting practices or improper storage conditions prior to freezing [35]. In terms of ripeness, there were cases of both overripe and unripe vegetables. Carrots and cauliflower were mainly overripe, while broccoli in some samples was too young at the moment of freezing, which could affect its structure and color after the process (Figure 2F). One of the blends did not meet the quality classification requirements for this criterion due to an excessive proportion of overripe vegetables at the rate of 11.99% (Figure 3c). The ripeness strongly affects freeze-thaw behavior: immature tissues tend to be pale and overly firm; overripe tissues soften excessively and may darken [36]. Maturity deviations impact sensory acceptability and may indicate poor raw material selection and inconsistent supplier control.

Analyzing the results of inedible residues, fragments of peel (especially in carrots) and the ends of pods in green beans were observed, which should have been removed during the preparation of the raw material. In most samples, no significant amounts of this were detected, and they were classified as Extra Class quality. Only one of the blends was classified as Class II with a higher number of inedible fragments (Figure 3d). Figure 2D shows examples of green bean pods found in one of the blends. These results are consistent with the commercial standard, which only specifies that inedible residues in quantities that affect commercial quality are unacceptable, and no such excesses have been recorded [29]. However, this highlights the need to improve trimming controls.

In addition to the quality parameters discussed above, other characteristics of frozen vegetables described in the standard were also checked, i.e., vegetable sizes (cubes, slices), vegetable firmness before and after thawing, and enzymatic activity. These parameters were within the standard for all tested samples of frozen vegetable blends. To summarize, the samples tested were characterized by quality variability. Despite maintaining basic safety parameters, in many cases, technological, visual, and raw material defects were observed, which may result from insufficient raw material selection, suboptimal blanching, or incorrect freezing and storage processes.

## 4. Conclusions

This study provides a comprehensive evaluation of the physicochemical composition, color stability, technological quality, and safety-relevant attributes of commercially available frozen vegetable blends. The results clearly indicate that the chemical integrity of the vegetables (including pH, acidity, moisture content, and texture) remains well preserved during industrial freezing. Similarly, color parameters matched those of fresh or properly blanched vegetables, suggesting that enzymatic inactivation and pigment stability were appropriately maintained in most products. These findings confirm that commercial freezing, when performed correctly, ensures high nutritional and chemical safety of vegetable components.

In contrast, significant discrepancies were observed in technological quality, with numerous blends failing to meet the requirements of PN-A-78608. Mechanical fragmentation, clumping, frostbite, color defects, diseased tissues, ripeness inconsistencies, and trimming residues were all detected. Importantly, several of these defects, particularly clumping and health condition defects, may represent indicators of potential safety-relevant issues.

## Figures and Tables

**Table 1:** Characteristics of the tested frozen vegetable blends-examples of different variants.

Type of Blend	Weight [g]	Packaging Material	Expiry Date	Composition According to the Producer
Pan-fried vegetables	450	foil not specified	04/2027	vegetables in varying proportions: carrots, green beans, corn, green peas, peppers, blanched red beans, onions
	750	LDPE4 foil	04/2027	vegetables in varying proportions 60% (green peas, brussels sprouts, chopped green beans, cauliflower, parsley), carrots 30%, celery 5%, leeks 5%
	1000	foil not specified	01/2027	tomato 35%, eggplant 21%, zucchini 20%, pepper 14% (red and green), onion 10%
Peas and carrots	450	foil not specified	02/2027	carrots 70%, green peas 30%
	450	LDPE4 foil	02/2027	vegetables in varying proportions 100% (carrots, peas)
	2500	foil not specified	01/2027	carrots 80%, green peas 20%
Vegetable bouquets	450	LDPE4 foil	08/2026	vegetables in varying proportions: cauliflower, green beans, carrots, brussels sprouts, green peas
	2000	LDPE4 foil	09/2026	vegetables in varying proportions: carrots, cauliflower, broccoli
	2500	foil not specified	11/2026	vegetables in varying proportions (carrot slices, cauliflower florets, broccoli florets)
Carrot mix Soup mix	300	07 other foil	11/2025	colorful carrots in varying proportions (red carrots, yellow carrots, white carrots, purple carrots)
	450	07 other foil	01/2027	vegetables in varying proportions 86% (carrot cubes, green beans, kohlrabi, cauliflower, brussels sprouts), leek slices 7%, celery cubes 7%
	750	LDPE4 foil	09/2026	vegetables in varying proportions: diced potatoes, diced carrots, cauliflower florets, brussels sprouts, diced onions, diced parsley root

**Table 2:** Results of physicochemical parameters for individual vegetables as mean ± standard deviation.

Vegetable	pH [–]	Total Acidity [g/100 g] ^1^	Water Content [%]	Dry Matter Content [%]	Hardness [N]
Broccoli	6.96 ± 0.22	0.060 ± 0.017	91.43 ± 1.06	8.57 ± 1.06	6.44 ± 2.82
Brussels sprouts	6.50 ± 0.11	0.117 ± 0.034	86.27 ± 1.23	13.73 ± 1.23	17.49 ± 14.84
Carrot	6.57 ± 0.77	0.049 ± 0.018	90.11 ± 1.44	9.91 ± 1.45	15.34 ± 18.25
Cauliflower	6.76 ± 0.25	0.053 ± 0.014	93.11 ± 0.43	6.89 ± 0.43	7.60 ± 5.57
Celery	6.48 ± 0.73	0.089 ± 0.030	91.18 ± 2.66	8.82 ± 2.66	12.71 ± 18.35
Chickpeas	6.59 ± 0.01	0.098 ± 0.005	57.03 ± 0.45	42.97 ± 0.45	99.98 ± 10.11
Corn	7.46 ± 0.02	0.034 ± 0.002	76.68 ± 0.10	23.32 ± 0.1	74.94 ± 26.27
Eggplant	4.72 ± 0.01	0.132 ± 0.013	93.24 ± 0.13	6.76 ± 0.13	7.47 ± 2.37
Green beans	6.38 ± 0.20	0.060 ± 0.022	88.02 ± 1.66	11.98 ± 1.66	8.84 ± 7.17
Green peas	6.87 ± 0.12	0.077 ± 0.019	72.00 ± 3.98	27.14 ± 4.62	34.06 ± 12.21
Kohlrabi	6.54 ± 0.02	0.122 ± 0.026	93.85 ± 0.03	6.15 ± 0.03	11.98 ± 2.22
Leeks	5.69 ± 0.13	0.100 ± 0.027	90.89 ± 0.96	9.11 ± 0.96	6.54 ± 3.98
Onion	5.61 ± 0.35	0.126 ± 0.020	91.88 ± 0.68	8.12 ± 0.68	6.38 ± 2.27
Parsley	6.52 ± 0.01	0.097 ± 0.006	88.21 ± 0.00	11.79 ± 0.00	9.41 ± 2.18
Parsnips	6.43 ± 0.01	0.059 ± 0.004	91.09 ± 0.04	8.91 ± 0.04	12.06 ± 0.77
Pepper	5.37 ± 0.17	0.134 ± 0.014	92.05 ± 0.96	7.95 ± 0.96	8.12 ± 6.00
Potato	6.25 ± 0.03	0.116 ± 0.006	82.25 ± 0.18	17.75 ± 0.18	13.45 ± 1.65
Red beans	6.39 ± 0.01	0.103 ± 0.009	59.07 ± 0.30	40.93 ± 0.30	99.28 ± 15.29
Tomato	4.40 ± 0.04	0.322 ± 0.009	93.66 ± 0.23	6.34 ± 0.23	8.13 ± 2.79
White turnip	6.51 ± 0.01	0.067 ± 0.005	93.72 ± 0.05	6.28 ± 0.05	8.13 ± 2.79
Zucchini	6.00 ± 0.08	0.058 ± 0.007	94.98 ± 0.14	5.02 ± 0.14	6.88 ± 1.28

^1^ Calculated as acetic acid.

**Table 3:** Results of color parameters for individual vegetables are shown as mean ± standard deviation.

Vegetable	Color Parameter				
	L* (D65)	a* (D65)	b* (D65)	C* (D65)	h (D65)
Broccoli	43.23 ± 7.73	−8.71 ± 1.65	19.36 ± 6.03	21.38 ± 5.71	115.69 ± 6.99
Brussels sprouts	46.27 ± 6.27	−6.31 ± 1.60	22.94 ± 5.18	23.86 ± 5.09	105.97 ± 5.19
Carrot	51.91 ± 1.91	30.57 ± 3.47	32.48 ± 3.08	44.65 ± 4.14	46.79 ± 2.72
Cauliflower	67.47 ± 5.48	−1.60 ± 0.92	16.80 ± 2.94	16.92 ± 2.88	95.85 ± 4.04
Celery	68.05 ± 2.10	0.44 ± 0.61	23.30 ± 3.45	23.31 ± 3.45	88.80 ± 1.41
Chickpeas	60.86 ± 0.12	9.09 ± 0.04	26.63 ± 0.18	28.14 ± 0.18	71.15 ± 0.05
Corn	65.79 ± 1.33	12.16 ± 0.17	40.64 ± 2.37	42.43 ± 2.28	73.30 ± 0.9
Eggplant	42.39 ± 0.19	8.75 ± 0.12	18.48 ± 0.13	20.45 ± 0.09	64.67 ± 0.41
Green beans	43.29 ± 2.30	−9.48 ± 1.86	16.09 ± 3.16	18.68 ± 3.64	120.57 ± 1.35
Green peas	48.27 ± 1.67	−10.20 ± 1.06	19.82 ± 1.53	22.31 ± 1.75	117.22 ± 1.65
Kohlrabi	66.37 ± 0.91	−1.53 ± 0.23	14.54 ± 0.75	14.62 ± 0.75	96.03 ± 0.97
Leeks	53.20 ± 4.98	−7.73 ± 1.04	20.16 ± 3.25	21.61 ± 3.30	111.18 ± 2.33
Onion	59.24 ± 2.36	−0.09 ± 2.18	16.66 ± 5.49	16.79 ± 5.51	91.69 ± 7.28
Parsnips	67.12 ± 0.09	−0.59 ± 0.04	20.53 ± 0.10	20.54 ± 0.10	91.65 ± 0.13
Pepper	38.36 ± 2.88	18.12 ± 12.13	17.70 ± 4.20	27.45 ± 6.92	51.66 ± 29.32
Potato	77.31 ± 0.13	0.07 ± 0.14	29.38 ± 0.31	29.38 ± 0.31	89.86 ± 0.27
Tomato	42.84 ± 0.08	23.41 ± 0.05	28.52 ± 0.14	36.90 ± 0.14	50.62 ± 0.07
White turnip	63.19 ± 0.63	−0.50 ± 0.06	14.42 ± 0.83	14.43 ± 0.83	92.01 ± 0.37
Zucchini	53.21 ± 10.50	−3.78 ± 1.20	19.61 ± 6.48	20.11 ± 6.09	102.94 ± 7.48

**Table 4:** Selected quality parameters of frozen vegetable blends (based on PN-A-78608).

Feature	Quality Class			
	Extra	I	II	Description
Crushed/mechanically damaged vegetables (%)	≤2	≤4	≤8	excessive fragmentation indicates mechanical damage during processing or transport
Clumped pieces (%)	≤3	≤7	≤10	reflect improper freezing or temperature fluctuations
Color defects (%)	≤2	≤10	≤15	unusual for the variety; brown, black, light discoloration
Health condition (pcs/500 g)	≤2	≤6	≤6	presence of vegetable components with disease changes or damaged by pests
Organic pollutants of plant origin (%)	≤0.1	≤0.2	≤0.3	plant-derived impurities (e.g., stems, leaves, seeds)
Residues of peel/inedible ends (pcs/500 g)	≤1	≤4	≤8	peel fragments, stalk ends and other non-edible vegetable parts
With frostbite (%)	≤2	≤4	≤10	vegetables showing surface frost damage (freezer burn), visible as dehydrated, whitish or toughened areas
Overripe/unripe (%)	≤1	≤3	≤10	vegetables that were visibly overmature (soft, discolored, fibrous) or immature (underdeveloped, pale, lacking typical texture)

## Data Availability

The key data generated and used to support the findings of this study are presented in this article. The remaining detailed datasets are available from the corresponding author upon reasonable request.
